# A Systematic Review of Natural Language Processing Methods and Applications in Thyroidology

**DOI:** 10.1016/j.mcpdig.2024.03.007

**Published:** 2024-05-21

**Authors:** Ricardo Loor-Torres, Mayra Duran, David Toro-Tobon, Maria Mateo Chavez, Oscar Ponce, Cristian Soto Jacome, Danny Segura Torres, Sandra Algarin Perneth, Victor Montori, Elizabeth Golembiewski, Mariana Borras Osorio, Jungwei W. Fan, Naykky Singh Ospina, Yonghui Wu, Juan P. Brito

**Affiliations:** Knowledge and Evaluation Research Unit (R.L.-T., M.D., M.M.C., C.S.., D.S.T., S.A.P., V.M., E.G., M.B.O., J.P.B.), Division of Endocrinology, Diabetes, Metabolism, and Nutrition (D.T.-T., J.P.B.), Department of Medicine, and Department of Artificial Intelligence and Informatics (N.S.O.), Mayo Clinic, Rochester, MN; University of Edinburgh, Edinburgh, Scotland, United Kingdom (D.S.T.); Montefiore Health Center, Albert Einstein College of Medicine, New York, NY (J.W.F.); Division of Endocrinology, Department of Medicine (N.S.O.), and Department of Health Outcomes and Biomedical Informatics (Y.W.), University of Florida, Gainesville, FL; and Respiratory, Cardiovascular, and Renal Pathobiology and Bioengineering, Universitat de Barcelona, Spain (D.S.T.).

## Abstract

This study aimed to review the application of natural language processing (NLP) in thyroid-related conditions and to summarize current challenges and potential future directions. We performed a systematic search of databases for studies describing NLP applications in thyroid conditions published in English between January 1, 2012 and November 4, 2022. In addition, we used a snowballing technique to identify studies missed in the initial search or published after our search timeline until April 1, 2023. For included studies, we extracted the NLP method (eg, rule-based, machine learning, deep learning, or hybrid), NLP application (eg, identification, classification, and automation), thyroid condition (eg, thyroid cancer, thyroid nodule, and functional or autoimmune disease), data source (eg, electronic health records, health forums, medical literature databases, or genomic databases), performance metrics, and stages of development. We identified 24 eligible NLP studies focusing on thyroid-related conditions. Deep learning-based methods were the most common (38%), followed by rule-based (21%), and traditional machine learning (21%) methods. Thyroid nodules (54%) and thyroid cancer (29%) were the primary conditions under investigation. Electronic health records were the dominant data source (17/24, 71%), with imaging reports being the most frequently used (15/17, 88%). There is increasing interest in NLP applications for thyroid-related studies, mostly addressing thyroid nodules and using deep learning-based methodologies with limited external validation. However, none of the reviewed NLP applications have reached clinical practice. Several limitations, including inconsistent clinical documentation and model portability, need to be addressed to promote the evaluation and implementation of NLP applications to support patient care in thyroidology.

Artificial intelligence (AI) aims to achieve human-like intelligence through entities (eg, machines), capable of processing and performing actions akin to human behavior.^1^ In addition, machine learning stands as a rapidly evolving field of computer science that seeks to train machines using data sets to perform time-consuming tasks that typically require human cognitive abilities. Its potential to solve real-world challenges across various domains, including health care, has led to increased research into its uses.^2^ By integrating different AI modalities into the decision-making process of physicians, this technology has the potential to enhance the field of medicine by improving the accuracy of diagnostic tests, streamlining provider workflow, enabling better disease and therapeutic monitoring, and resulting in better patient outcomes.^3,4^

Natural language processing (NLP) represents the intersection between linguistics and AI, analyzing text and speech to achieve human-like language understanding.^5^ Despite its more than 50-year history, the growing applicability in medicine has sparked significant interest in these technologies, particularly by overcoming previous limitations through advancements in machine and deep learning.^5,6^ In health care, NLP models have primarily been used to identify and extract information from unstructured data from the electronic health records (EHRs).^7^ The implementation of EHRs has led to exponential growth in the volume of health care data over the past 2 decades. However, only around 20% of EHR data are estimated to be structured as diagnostic or billing codes and simple clinical variables like vital signs or laboratory results. In contrast, the bulk of data in EHRs are unstructured in free text, like clinical notes or diagnostic reports, making their use in research time-consuming and challenging.^8^ To address this challenge, NLP techniques have been developed to efficiently extract and standardize diverse medical data from textual sources, encompassing elements like history and physical examinations, laboratory findings, diagnostic reports, and treatment records.^9^ For example, in radiology, NLP has been used to identify specific features of interest within imaging reports.^10^ Similarly, in the field of oncology, it has been leveraged to extract and categorize information from pathology reports, thereby supporting the staging and prediction of outcomes for various types of cancer.^11^

Thyroid disorders are highly prevalent among the general population. Recent studies have demonstrated the effectiveness of various NLP systems in extracting relevant information from EHRs within the field of thyroidology.^12^ These NLP-driven approaches have shown promise in enhancing and validating diagnostic and prognostic tools for various thyroid pathologies, including functional thyroid disorders, thyroid nodules, and thyroid cancer.^4,13^ Despite these promising developments, the existing literature lacks a comprehensive overview. Such an overview could prove invaluable for clinicians, researchers, and other stakeholders interested in understanding how NLP can be applied to improve the care of patients with thyroid diseases. This study was designed to fill this gap by systematically reviewing the applications of NLP in thyroid-related conditions. We also aimed to summarize the current challenges and provide insights into future perspectives in this burgeoning field.

## METHODS

This systematic review was conducted based on a priori established protocol, available in PROSPERO (CRD42022375085). This article is reported according to the Preferred Reporting Items for Systematic Reviews and Meta-Analysis guidelines (PRISMA).^14^

### Data Sources and Search Strategy

A comprehensive search was conducted in multiple scientific databases for articles published between January 2012 and November 2022. Databases included Ovid MEDLINE, Epub Ahead of Print, In-Process & Other Non-Indexed Citations; Daily; Ovid EMBASE; Ovid Cochrane Central Register of Controlled Trials; Ovid Cochrane Database of Systematic Reviews; and Scopus. The search strategy for references using NLP in patients with thyroid conditions was designed and conducted by an experienced librarian (L.P.). Subsequently, a snowballing technique was applied to expand our search by identifying additional potentially relevant references that might have been overlooked during the initial search or published after our search timeline, until April 2023. The full search strategy is described in Supplemental Material (available online at https://www.mcpdigitalhealth.org/).

### Eligibility Criteria

We included studies describing empirical research about the application of NLP methods for any thyroid-related condition. Studies were excluded for the following reasons: nonempirical works, such as editorials, systematic reviews, and commentaries; not performed in humans; or not published or available in English. In cases of multiple studies by the same research group using the same data set, we prioritized based on the publication date (including the most recent and excluding the earlier ones).

### Study Selection Process

All titles, abstracts, and full texts were independently screened using standardized and piloted criteria through the Distiller Systematic Review software by pairs of reviewers (D.S.T., C.S.J., M.D., and R.L.-T.). For each stage, we conducted pilots to calibrate judgments among the reviewers. Disagreements between 2 reviewers were resolved by consensus.

### Data Collection and Analyses

For all included studies, we extracted the following variables: general study characteristics, including year, country, and population demographic characteristics; and NLP applications or tasks (eg, identification, classification, or automation). Notably, certain publications involved multiple NLP applications, which were categorized accordingly. Furthermore, we noted the thyroid conditions (eg, thyroid cancer, thyroid nodule, or functional or autoimmune disease), data source (eg, EHR, online health forums or social media, medical literature databases, or genomic databases), evaluated data set, applied NLP methods (eg, rule-based, machine learning, deep learning, or hybrid), performance metrics (eg, accuracy, sensitivity [SN], and positive predictive value [PPV]), and stages of development of the models (eg, training, validation, or testing). In cases in which the data were unavailable, we abstained from extraction.

Results were summarized using frequencies and percentages for categorical variables. The exploratory nature of the research question, the heterogeneity of methodology, and the lack of consensus on the quality assessment approach for the NLP models prevented a quantitative meta-analysis and the evaluation of the risk of bias. Instead, data were analyzed using a meta-narrative approach.^15^

## RESULTS

### Study Selection and Characteristics

**FIGURE 1.**
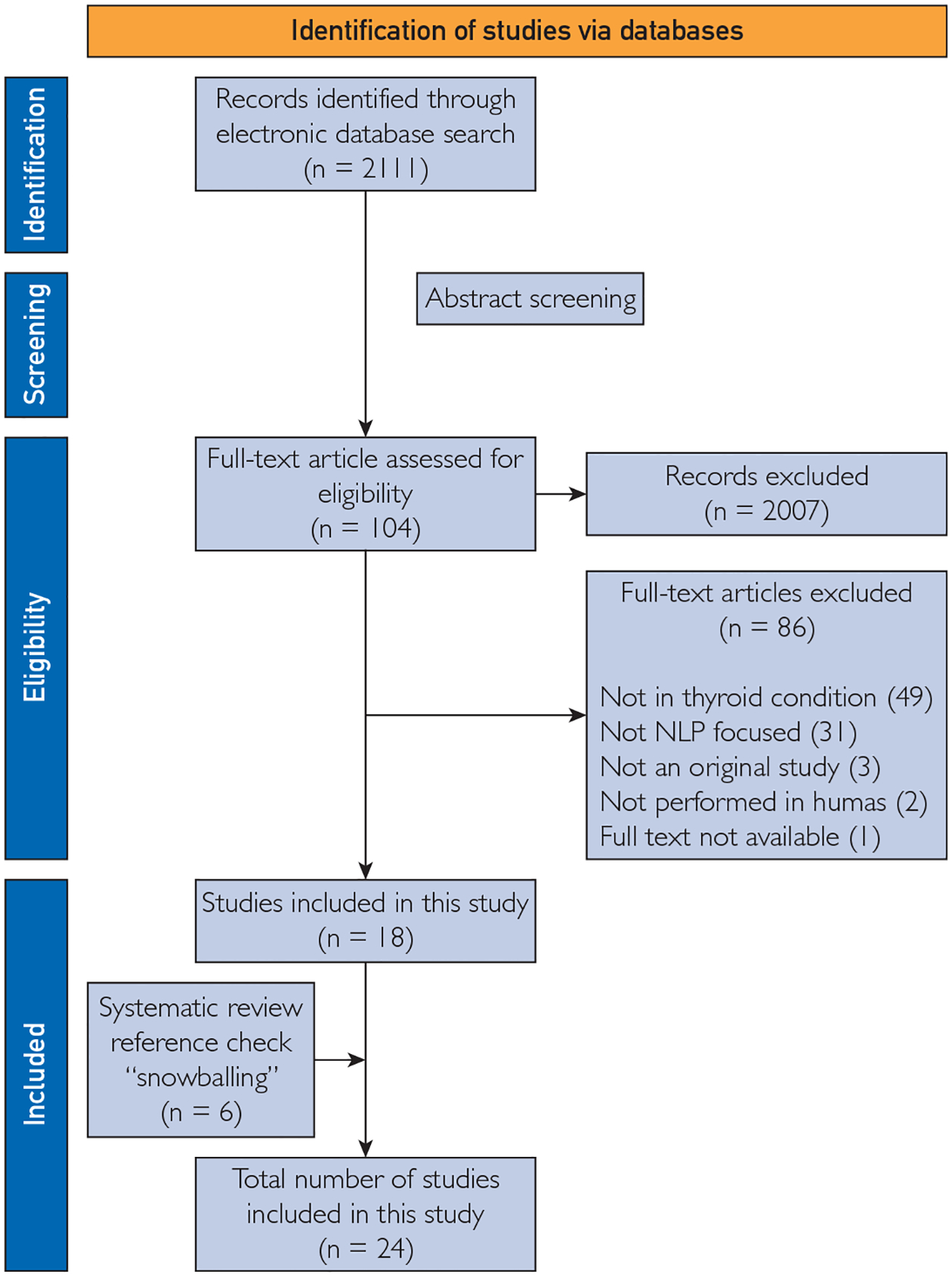
PRISMA-P flow diagram depicting the study selection process. NLP, natural language processing; PRISMA, Preferred Reporting Items for Systematic Reviews and Meta-Analysis.

A total of 2111 potentially relevant references were identified and retrieved through the electronic database search. After screening the titles and abstracts, 2007 studies were excluded. During the subsequent full-text screening, 86 studies were excluded, primarily for either not describing applied NLP methods or not focusing on thyroid conditions. Ultimately, 18 articles met our eligibility criteria and were included in the review. Using the snowballing technique, we identified and added 6 relevant studies to our analysis. Consequently, 24 articles were finally included in our study (Figure 1).

An increasing trend in yearly NLP-based thyroid publications was observed, especially in the past 3 years, with more than half of the included articles falling within this time-frame (15/24, 62.5%). Most studies were conducted in the United States (12/24, 50%), followed by China (7/24, 29%) and South Korea (3/24, 12%). In addition, demographic characteristics were reported in approximately one-third of the studies included (9/24, 37%) (Table).^16–38^

### Data Sources

**FIGURE 2.**
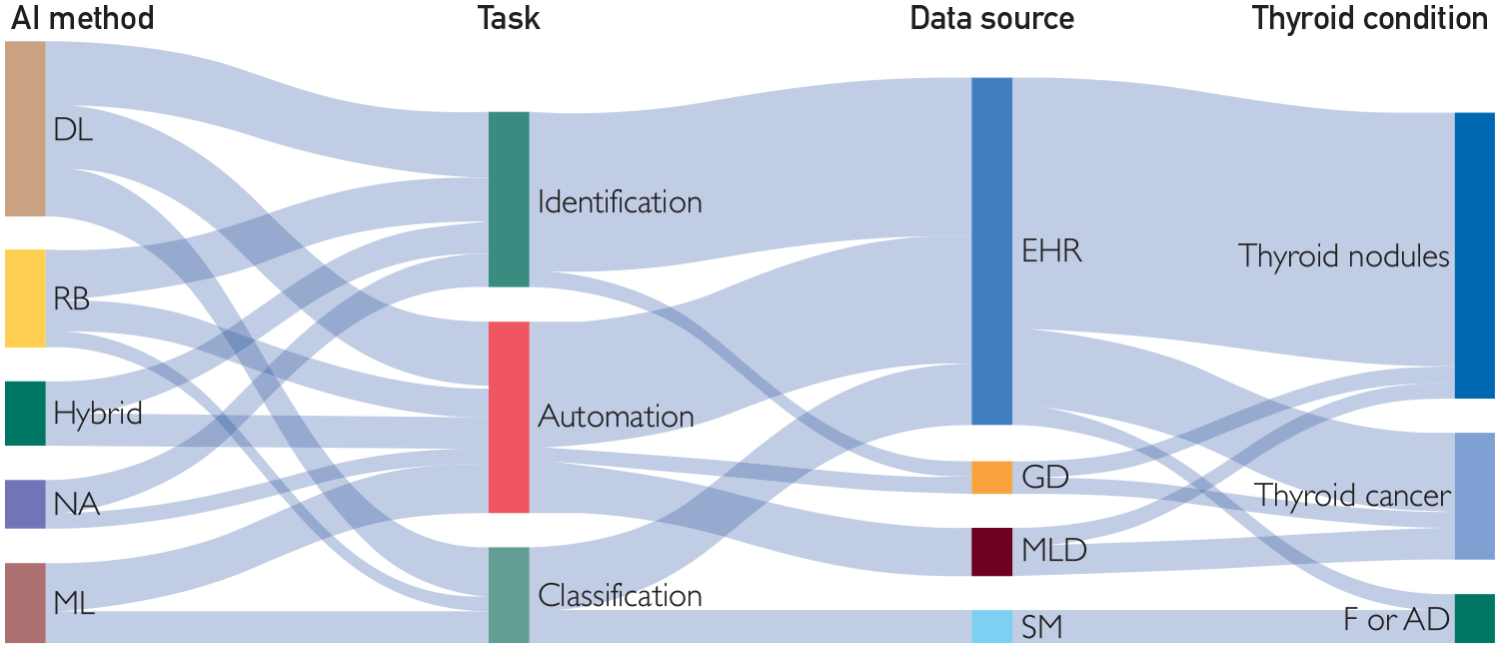
Sankey flow diagram depicting the distribution of thyroid. DL, deep learning; EHR, electronic health records; F or AD, functional and autoimmune disease; GD, genetic database; ML, machine learning; MLD, medical literature database; NA, not applicable or reported; RB, ruled-based; SM, social media.

Most studies included EHR as the retrieved source (17/24, 71%), followed by medical literature databases (3/24, 13%) such as PubMed, genomic databases (2/24, 8%), and online health forums or social media (2/24, 8%) (Figure 2). Within the studies that used data from EHRs, radiologic reports emerged as the most used source, constituting 88% (15/17) of the cases. Among these, thyroid ultrasound data were the prevailing imaging modality, used in 73% (11/15) of instances, while computer tomography scan reports were used in 20% (3/15) of cases. Three studies used both radiology and pathology reports.^12,27,35^ The studies were presented with varying data set sizes. Of the 24 studies, 11 (45%) used sample sizes of less than 5000 records, and 9 of 24 (38%) studies used data sets with more than 10,000 records.

### NLP Application in Thyroid Conditions

We found that thyroid nodule was the most investigated domain (13/24, 54%), followed by thyroid cancer (7/24, 29%), and functional and autoimmune disease (4/24, 17%). Supplemental Table 1 (available online at https://www.mcpdigitalhealth.org/) describes an in-depth analysis of the NLP methods used on various data sources to achieve distinct tasks within the thyroid conditions Supplemental Table 2.

#### NLP Applications in Thyroid Nodules.

Several NLP algorithms were developed and trained on large data sets to classify nodules as benign or malignant based on reported radiologic features (accuracy, 86%−88%; SN, 84%−92%; and PPV, 94%).^18,38^ In addition, other NLP models aimed at identifying granular radiology characteristics from ultrasound reports (accuracy, 77%−98%; SN, 85%−98%; and PPV:,74%−98%).^21,30,31^

Incidental thyroid findings are often observed in nonthyroid-related images, leading to increased detection of nodules and a potential thyroid cancer diagnosis. Drake et al^22^ used an NLP algorithm to evaluate the prevalence of incidental findings across various imaging modalities. In addition, Canton et al^16^ developed a highly accurate model for detecting thyroid lesions in imaging studies frequently used during trauma assessments in emergency department settings (SN, 90%; specificity, 95.3%).

The Thyroid Imaging, Reporting, and Data System (TI-RADS) is commonly used to ensure consistency in reporting systems for thyroid nodule characteristics.^39,40^ Chen et al^19^ developed a model to capture the crucial missing elements of TI-RADS. Moreover, Short et al^32^ engineered an NLP pipeline tailored to identify radiologic reports that align with the criteria for follow-up assessments. Their model exhibited promising accuracy (96.5%), SN (92.1%), and specificity (96%).Furthermore, Santos et al^31^ introduced a model designed to integrate TI-RADS reports with patient demographic information and comorbidities. Significantly, this model underwent prospective validation (accuracy, 0.89; F1 score, 0.99) and testing at an external facility (accuracy, 0.85; F1 score, 0.94).^31^ Finally, Zhang et al^35^ crafted a multistep model capable of integrating data from thyroid images, pathology reports, and radiology reports, achieving a commendable accuracy rate of 83%.

#### NLP Applications in Thyroid Cancer.

Lian et al^25^ developed an NLP pipeline to measure and categorize health-related quality of life based on narrative interviews with patients who underwent surgical treatment (area under the curve, 0.76; accuracy, 70.09; SN, 70.02%; and PPV, 70.20%).^25^ In addition, Yoo et al^12^ developed an NLP algorithm to determine thyroid cancer diagnosis and stage based on retrospective information from medical records, specifically using surgical pathology and whole-body scan reports (SN, 100%; PPV, 100%). Another application was able to retrieve information from online publications and genomic databases to identify genes contributing to nonmedullary thyroid cancer, encompassing 95%−97% of all thyroid cancers. The study by Zhou et al^37^ used an NLP algorithm to identify nonmedullary thyroid cancererelated genes from online databases.

#### NLP Applications in Functional and Autoimmune Disease.

Grani et al^23^ used an NLP pipeline to collect data from text messages in an online open medical forum to analyze patient experiences of living with hypothyroidism and concerns regarding their medication. Similarly, Park and Hong^28^ aimed to uncover patients’ perspectives on thyroid hormone replacement therapy from online health forums (WebMD) and determine its impact on treatment satisfaction. In another study, Zheng et al^36^ developed an NLP-based tool, capable of extracting clinical characteristics of patients with hypothyroidism from EHRs based on phenotypes collected from an array of medical resources, with an accuracy rate of over 97%.^36^ Finally, Luft et al^26^ used NLP to extract clinical features from EHRs from a cohort of pediatric patients with mood and anxiety disorders and correlated them with abnormal thyroid stimulating hormone levels.

## DISCUSSION

The digitalization of health care has aimed to improve patient care, streamline health care processes, and revolutionize clinical and health care delivery research.^41^ NLP can retrieve large volumes of narrative data and transform it into computable elements for downstream analyses, which was previously limited by labor-intensive and time-consuming manual extraction by human annotators.^42^ As in every area of health care, the potential applications and benefits of NLP in thyroid diseases are enormous. For instance, we noted an increasing number of NLP-based thyroid publications, especially in the past 3 years, with more than half of the included articles falling within this timeframe. To our knowledge, this is the first systematic review of NLP applications in the context of thyroidology. Our systematic review identified that, although still limited, NLP is already being used to study thyroid diseases, particularly thyroid nodules, and thyroid cancer, which were the focus of 54% and 29% of the studies included in our review, respectively.

Studies have applied NLP to extract thyroid nodule features from radiology reports leveraging state-of-the-art deep learning models.^30^ Furthermore, although some models focused on basic tasks, such as detecting the presence of thyroid incidentalomas in computed tomography, magnetic resonance imaging, or ultrasound reports,^16,22^ more sophisticated models were capable of determining whether the reported incidentaloma met the criteria for further ultrasound evaluation and additionally tracked the completion of such evaluations.^32^ Compared with traditional manual extraction of unstructured data, these models effectively harnessed large volumes of data, facilitating observational research, promoting quality improvement initiatives, enabling standardization of unstructured documentation, and developing real-time predictive tools for clinical care. In addition, NLP models were able to build large-volume data sets by extracting features from different free text sources, including diagnostic and pathology reports and clinician documentation.^12,21^ Furthermore, some models standardized unstructured data from multiple institutions to generate multicenter data sets.^12,19,21,29,34^

Although most models used EHR data, other valuable data sources have been explored. Zheng et al^36^ used NLP-extracted phenotypes from several online resources. Others used NLP tools to facilitate abstract screening for systematic literature reviews on thyroid cancer genetic associations or even automatically analyzing and integrating findings from previously published literature.^24^ These initiatives allowed the researchers to use data more efficiently and comprehensively from the exponentially growing body of literature and genetic repositories. In addition, Park and Hong^28^ used NLP on social media posts to identify issues related to thyroid hormone replacement from patient medication reviews. This type of tool can provide a more complete picture of the patient experience, including their emotional and social responses to their condition and treatments, which might not be efficiently captured in clinical records, to facilitate the identification of themes for discussion during shared decision-making encounters and characterize elements to be considered on future treatment or quality-of-life studies.

Notwithstanding the promising results of NLP, none of the applications included in this review are readily available for use in clinical practice. Notably, among the 24 studies identified, only 1 adopted a prospective design,^31^ and 2 undertook validation of their NLP models in an external health care setting.^31,34^ The road to incorporating these NLP interventions into routine research or clinical practice is riddled with several challenges that need careful consideration and concerted efforts to surmount. Specifically in the thyroid field, we hypothesize that the uptake of NLP methods is associated with the complexity of the thyroid-related domains, variations in language expression and reporting styles, completeness and accuracy of clinical documentation (ie, data on patient-specific concerns, complaints, or severity of symptoms depends on the accuracy of providers’ documentation), semantic (ie, misspellings, abbreviations, acronyms, or synonyms), and context (ie, it is challenging to create algorithms that can appropriately extract chronologic descriptions or simultaneous references in situations like a thyroid ultrasound report that includes several nodules), which can affect the NLP outcome, decrease the performance of the algorithm when applied to different institutions, and limit the portability and scalability of the interventions.^7,21,34,43^ In addition, data sources need to be representative of the population to avoid the incorporation of inequities and social bias into the models.^44^ Finally, using NLP methods requires high optimization for the local environment and extensive domain knowledge, which can be expensive, and stakeholders’ lack of financial resources or prioritization could halt their implementation. Thus, efficacy and cost-effectiveness trials are important to demonstrate the value of the intervention and facilitate its adoption.^41^

The examination of NLP studies within the domain of thyroidology, as presented in this review, has brought to light a pronounced divergence in NLP methods. Deep learning emerged as the preferred NLP method, with some studies applying pretrained large language models,^16,25,30,34^ followed by rule-based approaches. However, it is noteworthy that some studies refrained from providing explicit specifications regarding the used NLP methods.

In contemplating the future of NLP within the domain of thyroid nodule and cancer management, it is evident that enhancing the reporting system methods and integrating models, particularly with the framework of large language models, will significantly expand the role of NLP. This integration marks a shift toward more sophisticated, efficient, and versatile applications of NLP in thyroid nodule and cancer management.

We acknowledge several limitations that should be considered when evaluating our results. The heterogeneity observed in methodology, outcomes, and performance metrics across the included studies made it unfeasible to consistently report the results of the individual studies and to conduct a metaanalysis. In addition, we must recognize the potential for publication bias, which could lead to an overrepresentation of favorable results. However, despite these limitations, it is crucial to highlight the strengths of our systematic review. We adhered diligently to the Preferred Reporting Items for Systematic Reviews and Meta-Analysis (PRISMA) statement guidelines, ensuring a methodical and standardized approach to our review process. Furthermore, we successfully identified many publications that met our rigorous inclusion criteria. Consequently, our findings provide a comprehensive and robust characterization of the current landscape of NLP in thyroidology, holding valuable implications for clinicians, researchers, and other stakeholders invested in this field of study.

## CONCLUSION

The utilization of NLP within the realm of thyroidology exhibits a growing interest and holds the potential for advancing both research and patient care, while mitigating the burden placed on healthcare system stakeholders. However, it is noteworthy that the domains of interest within thyroidology and the NLP methodologies used remain somewhat restricted. Consequently, ample opportunity exists for further exploration, encompassing the untapped potential of NLP applications in various thyroid conditions that have yet to be investigated.

## Supplementary Material

1

2

3

4

## Figures and Tables

**TABLE.** Baseline Characteristics of the Studies Analyzed in This Systematic Review

Reference, year	Study design	Demographic information^a^	Thyroid condition	Data source	Cohort	AI methods and tasks
Canton et al,^16^ 2021; USA	Single center Retrospective	Age, gender, ethnicity	Thyroid nodules	EHR Radiology reports	2289 records	DL Identification
Chen et al,^17^ 2017; China	Single center Retrospective	NA	Thyroid nodules	EHR Radiology reports	13,592 records	DL Classification
Chen et al,^18^ 2018; China	Single center Retrospective	NA	Thyroid nodules	EHR Radiology reports	6116 records	DL Classification
Chen et al,^19^ 2020; USA	Multicenter Retrospective	NA	Thyroid nodules	EHR Radiology reports	247 records	RB Identification, automation
Chen et al,^20^ 2022; China	NA Retrospective	NA	Thyroid nodules	MLD PubMed	5770 references	ML Automation
Dedhia et al,^21^ 2022; USA	Multicenter Retrospective	NA	Thyroid nodules	EHR Radiology reports	1612 records	Hybrid Identification, automation
Drake et al,^22^ 2019; USA	Multicenter Retrospective	Age, gender	Thyroid nodules	EHR Radiology reports	51,907 records	RB Identification
Grani et al,^23^ 2021; Italy	NA Retrospective	NA	Functional or autoimmune disease	SM Medicitalia.it	27,525 entries	ML Classification
Kongburan et al,^24^ 2016; Thailand	NA Retrospective	NA	Thyroid cancer	MLD PubMed	500 references	ML Automation
Lian et al,^25^ 2023; USA	Single center Retrospective	NA	Thyroid cancer	EHR Interviews	500 records 100 patient sample	DL Classification
Luft et al,^26^ 2019; USA	Single center Retrospective	Age, gender, ethnicity	Functional or autoimmune disease	EHR Patient charts	84,000 records 1319 patient sample	RB^b^ Identification
Miao et al,^27^ 2020; China	Single center Retrospective	Age, gender	Thyroid nodules	EHR Radiology and pathology reports	1290 records	NA Identification
Park and Hong,^28^ 2018; South Korea	NA Retrospective	Age, gender	Functional or autoimmune disease	SM WebMD	1768 entries	ML Classification
Park et al,^29^ 2021; South Korea	Multicenter Retrospective	Age, gender	Thyroid cancer	EHR Radiology reports	308 records 220 patient sample	RB Automation
Pathak et al,^30^ 2023; USA	Multicenter Retrospective	NA	Thyroid nodules	EHR Radiology reports	490 records	DL Identification
Santos et al,^31^ 2021; USA	Single center Retrospective and prospective	Age, gender, ethnicity	Thyroid nodules	EHR Radiology reports	1132 records	DL Identification, automation
Short et al,^32^ 2022; USA	Single center Retrospective	Age, gender, ethnicity, comorbidities	Thyroid nodules	EHR Radiology reports	13,385 records	DL Identification, automation
Yoo et al,^12^ 2022; South Korea	Multicenter Retrospective	Age, gender	Thyroid cancer	EHR Radiology and pathology reports	108,372 records	RB Classification
Zhang et al,^33^ 2021; China	NA Retrospective	NA	Thyroid cancer	MLD PubMed	34,692 references	ML Automation
Zhang et al,^34^ 2022; China	Multicenter Retrospective	NA	Thyroid cancer	HER Radiology reports	788,129 records	DL Automation
Zhang et al,^35^ 2023; USA	Multicenter Retrospective	NA	Thyroid nodules	HER Radiology and pathology reports	565 records 471 patient sample	Hybrid Identification, automation
Zheng et al,^36^ 2020; USA	NA Retrospective	NA	Functional or autoimmune disease	GD BioVU DNA biobank	772,394 records 84,821 patient sample	NA Identification
Zhou et al,^37^ 2021; USA	NA Retrospective	NA	Thyroid cancer	GD ClinGen, NCCN guidelines, OMIM, Genetics Home Reference, GeneCards, and Gene-NCBI	NA	NA Automation
Zou et al,^38^ 2021; China	Single center Retrospective	NA	Thyroid nodules	HER Radiology reports	5328 records	DL Classification

a Analysis from studies in which demographic information from data sets were reported.

b Not specified, however presumed to be the most likely implemented method.

DL, deep learning; EHR, electronic health records; GD, genetic database; ML, machine learning; MLD, medical literature database; NA, not applicable or reported; RB, ruled-based; SM, social media.

## ARTICLE HIGHLIGHTS

- NLP has the potential to streamline healthcare data retrieval, reducing laborious tasks for stakeholders.
- Despite its promising applications in research and patient care, our review revealed limited NLP exploration in thyroidology.
- The most investigated domain was thyroid nodules, primarily using electronic health records with radiology reports as the primary data source.
- Traditional challenges in NLP exploration and implementation include language diversity, contextual comprehension, and model portability.
- Large language models have been recently explored and offer solutions to previously NLP encountered challenges.

## Abbreviations and Acronyms:

AI: artificial intelligence
EHR: electronic health record
NLP: natural language processing
PPV: positive predictive value
SN: sensitivity
TI-RADS: Thyroid Imaging, Reporting, and Data System

## References

[1] Sarker IH. AI-based modeling: techniques, applications and research issues towards automation, intelligent and smart systems. SN Comput Sci. 2022;3(2):158. 10.1007/s42979-022-01043-x. 35194580 PMC8830986

[2] Silva GFS, Fagundes TP, Teixeira BC, Chiavegatto Filho ADP. Machine learning for hypertension prediction: a systematic review. Curr Hypertens Rep. 2022;24(11):523–533. 10.1007/s11906-022-01212-6. 35731335

[3] Kaul V, Enslin S, Gross SA. History of artificial intelligence in medicine. Gastrointest Endosc. 2020;92(4):807–812. 10.1016/j.gie.2020.06.040. 32565184

[4] Toro-Tobon D, Loor-Torres R, Duran M, Artificial intelligence in thyroidology: a narrative review of the current applications, associated challenges, and future directions. Thyroid. 2023;33(8):903–917. 10.1089/thy.2023.0132. 37279303 PMC10440669

[5] Nadkarni PM, Ohno-Machado L, Chapman WW. Natural language processing: an introduction. J Am Med Inform Assoc. 2011; 18(5):544–551. 10.1136/amiajnl-2011-000464. 21846786 PMC3168328

[6] Esteva A, Robicquet A, Ramsundar B, A guide to deep learning in healthcare. Nat Med. 2019;25(1):24–29. 10.1038/s41591-018-0316-z. 30617335

[7] Hossain E, Rana R, Higgins N, Natural language processing in electronic health records in relation to healthcare decisionmaking: a systematic review. Comput Biol Med. 2023;155: 106649. 10.1016/j.compbiomed.2023.106649. 36805219

[8] HIT Consultant. Why unstructured data holds the key to intelligent healthcare systems. https://hitconsultant.net/2015/03/31/tapping-unstructured-data-healthcares-biggest-hurdle-realized/.

[9] Demner-Fushman D, Chapman WW, McDonald CJ. What can natural language processing do for clinical decision support? J Biomed Inform. 2009;42(5):760–772. 10.1016/j.jbi.2009.08.007. 19683066 PMC2757540

[10] Mithun S, Jha AK, Sherkhane UB, Clinical concept-based radiology reports classification pipeline for lung carcinoma. J Digit Imaging. 2023;36(3):812–826. 10.1007/s10278-023-00787-z. 36788196 PMC10287609

[11] Yim WW, Yetisgen M, Harris WP, Kwan SW. Natural language processing in oncology: a review. JAMA Oncol. 2016;2(6):797–804. 10.1001/jamaoncol.2016.0213. 27124593

[12] Yoo S, Yoon E, Boo D, Transforming thyroid cancer diagnosis and staging information from unstructured reports to the observational medical outcome partnership common data model. Appl Clin Inform. 2022;13(3):521–531. 10.1055/s-0042-1748144. 35705182 PMC9200482

[13] Idarraga AJ, Luong G, Hsiao V, Schneider DF. False negative rates in benign thyroid nodule diagnosis: machine learning for detecting malignancy. J Surg Res. 2021;268:562–569. 10.1016/j.jss.2021.06.076. 34464894

[14] Shamseer L, Moher D, Clarke M, Preferred reporting items for systematic review and meta-analysis protocols (PRISMA-P) 2015: elaboration and explanation. BMJ. 2015; 350:g7647. 10.1136/bmj.g7647. 25555855

[15] Greenhalgh T, Robert G, Macfarlane F, Bate P, Kyriakidou O, Peacock R. Storylines of research in diffusion of innovation: a meta-narrative approach to systematic review. Soc Sci Med. 2005;61(2):417–430. 10.1016/j.socscimed.2004.12.001. 15893056

[16] Canton SP, Dadashzadeh E, Yip L, Forsythe R, Handzel R. Automatic detection of thyroid and adrenal incidentals using radiology reports and deep learning. J Surg Res. 2021;266: 192–200. 10.1016/j.jss.2021.03.060. 34020097

[17] Chen D, Shi C, Wang M, Pan Q. Thyroid nodule classification using hierarchical recurrent neural network with multiple ultrasound reports. In: Neural Information Processing; 24th International Conference, ICONIP 2017, Guangzhou, China, November 14–18, 2017, Proceedings, Part V 24. Springer; 2017:765–773.

[18] Chen D, Zhang J, Li W. Thyroid nodule classification using two levels attention-based bi-directional LSTM with ultrasound reports. In: 2018 9th International Conference on Information Technology in Medicine and Education (ITME). IEEE; 2018. 10.1109/ITME.2018.0007.

[19] Chen KJ, Dedhia PH, Imbus JR, Schneider DF. Thyroid ultrasound reports: will the thyroid imaging, reporting, and data system improve natural language processing capture of critical thyroid nodule features? J Surg Res. 2020;256:557–563. 10.1016/j.jss.2020.07.015. 32799005 PMC8102071

[20] Chen P, Feng C, Huang L, Chen H, Feng Y, Chang S. Exploring the research landscape of the past, present, and future of thyroid nodules. Front Med (Lausanne). 2022;9:831346. 10.3389/fmed.2022.831346. 36714145 PMC9877524

[21] Dedhia PH, Chen K, Song Y, Ambiguous and incomplete: natural language processing reveals problematic reporting styles in thyroid ultrasound reports. Methods Inf Med. 2022;61(1–2): 11–18. 10.1055/s-0041-1740493. 34991173

[22] Drake T, Gravely A, Westanmo A, Billington C. Prevalence of thyroid incidentalomas from 1995 to 2016: a single-center, retrospective cohort study. J Endocr Soc. 2020;4(1):bvz027. 10.1210/jendso/bvz027. PMC6977946 31993553

[23] Grani G, Lenzi A, Velardi P. Supporting personalized health care with social media analytics: an application to hypothyroidism. ACM Trans Comput Healthcare. 2022;3(1):1–28. 10.1145/3468781.

[24] Kongburan W, Padungweang P, Krathu W, Chan JH. Semi-automatic construction of thyroid cancer intervention corpus from biomedical abstracts. In: 2016 Eighth International Conference on Advanced Computational Intelligence (ICACI). IEEE; 2016:150–157.

[25] Lian R, Hsiao V, Hwang J, Predicting health-related quality of life change using natural language processing in thyroid cancer. Intell Based Med. 2023;7:100097. 10.1016/j.ibmed.2023.100097. 37664403 PMC10473865

[26] Luft MJ, Aldrich SL, Poweleit E, Thyroid function screening in children and adolescents with mood and anxiety disorders. J Clin Psychiatry. 2019;80(5):18m12626. 10.4088/JCP.18m12626. 31390496

[27] Miao S, Jing M, Sheng R, The analysis of differential diagnosis of benign and malignant thyroid nodules based on ultrasound reports. Gland Surg. 2020;9(3):653–660. 10.21037/gs.2020.04.03. 32775255 PMC7347809

[28] Park SH, Hong SH. Identification of primary medication concerns regarding thyroid hormone replacement therapy from online patient medication reviews: text mining of social network data. J Med Internet Res. 2018;20(10):e11085. 10.2196/11085. 30355555 PMC6231751

[29] Park J, You SC, Jeong E, A framework (SOCRATex) for hierarchical annotation of unstructured electronic health records and integration into a standardized medical database: development and usability study. JMIR Med Inform. 2021;9(3): e23983. 10.2196/23983. 33783361 PMC8044740

[30] Pathak A, Yu Z, Paredes D, Extracting thyroid nodules characteristics from ultrasound reports using transformer-based natural language processing methods. AMIA Annu Symp Proc. 2023: 1193–1200. 10.48550/arXiv.2304.0011. 38222394 PMC10785862

[31] Santos T, Kallas ON, Newsome J, Rubin D, Gichoya JW, Banerjee I. A fusion NLP model for the inference of standardized thyroid nodule malignancy scores from radiology report text. AMIA Annu Symp Proc; 2021;2021:1079–1088. 35308953 PMC8861701

[32] Short RG, Dondlinger S, Wildman-Tobriner B. Management of incidental thyroid nodules on chest CT: using natural language processing to assess white paper adherence and track patient outcomes. Acad Radiol. 2022;29(3):e18–e24. 10.1016/j.acra.2021.02.019. 33757722

[33] Zhang Z, Yao L, Wang W, Jiang B, Xia F, Li X. A bibliometric analysis of 34,692 publications on thyroid cancer by machine learning: how much has been done in the past three decades? Front Oncol. 2021;11:673733. 10.3389/fonc.2021.673733. 34722236 PMC8551832

[34] Zhang Q, Zhang S, Li J, Improved diagnosis of thyroid cancer aided with deep learning applied to sonographic text reports: a retrospective, multi-cohort, diagnostic study. Cancer Biol Med. 2021;19(5):733–741. 10.20892/j.issn.2095-3941.2020.0509. 34491007 PMC9196053

[35] Zhang J, Mazurowski MA, Allen BC, Wildman-Tobriner B. Multistep Automated Data Labelling Procedure (MADLaP) for thyroid nodules on ultrasound: an artificial intelligence approach for automating image annotation. Artif Intell Med. 2023;141: 102553. 10.1016/j.artmed.2023.102553. 37295897

[36] Zheng NS, Feng Q, Kerchberger VE, PheMap: a multi-resource knowledge base for high-throughput phenotyping within electronic health records. J Am Med Inform Assoc. 2020; 27(11):1675–1687. 10.1093/jamia/ocaa104. 32974638 PMC7751140

[37] Zhou J, Singh P, Yin K, Non-medullary thyroid cancer susceptibility genes: evidence and disease spectrum. Ann Surg Oncol. 2021;28(11):6590–6600. 10.1245/s10434-021-09745-x. 33660127

[38] Zuo M, Zhao H, Huang M, Chen D. Knowledge-Powered Thyroid Nodule Classification with Thyroid Ultrasound Reports. In: 2021 IEEE International Conference on Dependable, Autonomic and Secure Computing, International Conference on Pervasive Intelligence and Computing, International Conference on Cloud and Big Data Computing, International Conference on Cyber Science and Technology Congress (DASC/PiCom/CBDCom/CyberSciTech). IEEE; 2021:597–604. 10.1109/DASC-PICom-CBDCom-CyberSciTech52372.2021.00103.

[39] Tessler FN, Middleton WD, Grant EG, ACR thyroid imaging, reporting and data system (TI-RADS): white paper of the ACR TI-RADS committee. J Am Coll Radiol. 2017;14(5):587–595. 10.1016/j.jacr.2017.01.046. 28372962

[40] Horvath E, Majlis S, Rossi R, An ultrasonogram reporting system for thyroid nodules stratifying cancer risk for clinical management. J Clin Endocrinol Metab. 2009;94(5):1748–1751. 10.1210/jc.2008-1724. 19276237

[41] Sharma A, Harrington RA, McClellan MB, Using digital health technology to better generate evidence and deliver evidence-based care. J Am Coll Cardiol. 2018;71(23):2680–2690. 10.1016/j.jacc.2018.03.523. 29880129

[42] Kim E, Rubinstein SM, Nead KT, Wojcieszynski AP, Gabriel PE, Warner JL. The evolving use of electronic health records (EHR) for research. Semin Radiat Oncol. 2019;29(4):354–361. 10.1016/j.semradonc.2019.05.010. 31472738

[43] Yang LWY, Ng WY, Foo LL, Deep learning-based natural language processing in ophthalmology: applications, challenges and future directions. Curr Opin Ophthalmol. 2021;32(5):397–405. 10.1097/ICU.0000000000000789. 34324453

[44] Newman-Griffis DR, Hurwitz MB, McKernan GP, Houtrow AJ, Dicianno BE. A roadmap to reduce information inequities in disability with digital health and natural language processing. PLoS Digit Health. 2022;1(11):e0000135. 10.1371/journal.pdig.0000135. 36812573 PMC9931310

