# Supplementary material for: A Systematic Review of Natural Language Processing Methods and Applications in Thyroidology
Source: Mayo Clin Proc Digit Health. Author manuscript; Available in PMC 2024 Jun 27. (PMC11210322; doi:10.1016/j.mcpdig.2024.03.007)
Supplement: 1 [file NIHMS2002602-supplement-1.pdf]

Supplemental Table. 1 Data sources, dataset sizes, and reported performance metrics of the NLP methods implemented in the selected references								
**Author/Year**	**Aim**	**Dataset**	**Accuracy**	**SN**	**SP**	**PPV**	**NPV**	**kappa**
**Thyroid nodules**								
Canton_2021	Identify thyroid and adrenal abnormalities present in CT scan reports	2289	-	0.9	0.95	-	-	-
Chen_2017	Classify thyroid nodules based on the characteristics found in ultrasound reports	13592	-	-	-	-	-	-
Chen_2018	Categorize thyroid nodule using ultrasound reports	6116	0.86	0.84	-	0.94	-	-
Chen_2020	Examine the ultrasound reporting quality and accuracy in extracting TI-RADS elements from ultrasound reports	247	-	-	-	-	-	-
Chen_2022	Evaluate the scope of thyroid nodule research over the previous 22 years	5770	-	-	-	-	-	-
Dedhia_2022	Extract granular features from ultrasound reports and uncover issues with the reporting language	1612	0.77	0.9	-	0.74	-	-
Drake_2019	Determine the prevalence of thyroid incidental findings across a diverse array of imaging techniques	51907	-	-	-	-	0.996	-

Miao_2020	Determine the use of ACR TI-RADS for classification of thyroid nodules as benign or malignant	1290	-	-	-	-	-	-
Pathak_2023	Extract granular features of thyroid nodules from ultrasound reports using five state-of-the-art NLP models	490	-	0.97	-	0.92	-	-
Santos_2021	Streamline the integration of information from TI-RADS with patient demographic data	1132	0.85-0.98	0.85-0.98	-	0.85-0.98	-	-
Short_2022	Explore adherence to ultrasonography follow-up recommendations for thyroid incidental findings and assess related downstream events	13385	0.96	0.92	0.96	0.45	0.99	-
Zhang_2023	Streamline the data labelling process of thyroid nodules by using pathology and radiology reports	565	0.83		-	-	-	-
Zou_2021	Evaluate thyroid nodule classification using ultrasound reports	5328	0.88	0-92	-	-	-	-

**Thyroid cancer**								
Kongburan_2016	Build databases of interventions for patients with thyroid cancer	500	-	-	-	-	-	-
Lian_2023	Identify Health Related Quality of Life from interviews of patients who underwent interventions for thyroid cancer	500	0.7	0.7		0.7	-	-
Park_2021	Develop a system to integrate standardized structured data with clinical documentation from medical records	308	-	-	-	-	-	-
Yoo_2022	Evaluate the applicability of diagnosing and staging thyroid cancer using the Observational Medical Outcome Partnership Common Data Model	108372	-	1	-	1	-	-
Zhang_2022	Assist radiologists in diagnosing thyroid cancer based on text reports.	788129	0.89-0.98	0.89-1.00	0.76-0.97	0.88-1.00	0.87-1.00	0.78-0.97
Zhang_2021	Provide a landscape of scientific publications on thyroid cancer."	34692	-	-	-	-	-	-
Zhou_2021	Conduct an NLP-based literature review focused on the association of genes with NMTC.	NA	-	-	-	-	-	-

**Functional and autoimmune diseases**								
Grani_2021	Describe patients' experiences with hypothyroidism and their treatment.	27525	0.84	0.86	-	0.83	-	-
Luft_2019	Determine abnormalities in TSH levels within hospitalized pediatrics patients with mood and anxiety disorders	84000	-	-	-	-	-	-
Park_2018	Describe patients' concerns regarding thyroid hormone replacement therapy and determine their satisfaction levels	1768	-	-	-	-	-	-
Zheng_2020	Optimize the phenotyping process for patients with hypothyroidism.	772394	-	-	-	-	-	-
**NPV: Negative predictive value, PPV: Positive predictive value, SN: Sensitivity, SP: Specificity**								
