# Supplementary material for: A Systematic Review of Natural Language Processing Methods and Applications in Thyroidology
Source: Mayo Clin Proc Digit Health. Author manuscript; Available in PMC 2024 Jun 27. (PMC11210322; doi:10.1016/j.mcpdig.2024.03.007)
Supplement: 2 [file NIHMS2002602-supplement-2.pdf]

Supplemental Table 2. Glossary of NLP methods and performance metrics.		
**Word**	**Definition**	**Ref.**
**Artificial intelligence concepts**		
Artificial intelligence (AI)	The simulation of human intelligence in computer systems and machines, aiming to perform specific tasks, including identification, classification, automation, and problem-solving.	3
Natural Language processing (NLP)	Subfield of AI focused on processing human language using algorithms and models to convert it into analyzable data.	3
Rule-based	Approach of AI based on predefined rules or logical statements to take decisions and execute tasks.	4
Machine learning (ML)	Subset of AI focused on algorithms and models that train the system to predict outcomes and learn from experience, patterns, and data, improving performance over time.	2, 3
Deep learning	Subfield of ML and AI used to model and solve complex problems with large amounts of data (including unlabeled and unstructured) using artificial neural networks with more than 3 layers (multi-layer). This approach is recommendable when dealing with large amounts of data and intricate patterns.	2, 3
Model	Mathematical or computational system trained with data to perform specific tasks.	1,3
Dataset	Data collections used to train, validate, and test AI algorithms.	4
Algorithm	List of detailed actions or steps within the instructions of the computer part of AI, designed to enable the performance of the tasks.	2, 3
Identification task	Process in NLP systems of recognizing and extracting specific pieces of information within text or data.	4
Classification task	NLP task which involves categorization of pieces of information based on its content, into the most appropriate predefined category.	4
Automation task	NLP process to systematize linguistic and textual processes.	4
Pipeline	Sequence of interconnected steps or processes applied to datasets to complete a task. Commonly used to streamline and automate complex processes in AI.	4
**Performance metrics**		
Accuracy	Metric used to estimate the performance of a classification model. Corresponds to the proportion of correctly classified observations.	5
Sensitivity (SN) - Recall	Measures the ability of the model to correctly identify true positive observations.	5
Specificity (SP)	Measures the capacity of the model to correctly not identify true negative observations.	5

Cohen's Kappa coefficient (Kappa)	Measures the agreement between two or multiple annotators when classifying data in class labels. This metric considers the effect of chance and gives a more robust measure of agreement than the regular percentage of agreement metric.	6
F1 score	Metric to evaluate classification task performance in imbalanced datasets, aiming to achieve a balance between precision and recall, while considering the trade-off between false positives and false negatives.	1, 4
Positive Predictive Value (PPV) - Precision	Proportion of true positives within all the positive results.	6
Negative Predictive Value (NPV)	Proportion of true negatives within all the negative results.	6

(1) Microsoft Glossary Definitions used in conversational language understanding - Azure AI services | Microsoft Learn

(2) Standford University AI-Definitions-HAI.pdf (stanford.edu)

(3) Mayo Clinic Glossary Glossary | Mayo Clinic Cloud

(4) Chat GPT

(5) Revision NLP heart Systematic review of current natural language processing methods and applications in cardiology (bmj.com)

(6) Epidemiology beyond the basics - 3rd edition - Moyses Szklo and Javier Nieto
