# Supplementary material for: A Systematic Review of Natural Language Processing Methods and Applications in Thyroidology
Source: Mayo Clin Proc Digit Health. Author manuscript; Available in PMC 2024 Jun 27. (PMC11210322; doi:10.1016/j.mcpdig.2024.03.007)
Supplement: 3 [file NIHMS2002602-supplement-3.pdf]

Ovid

Database(s): EBM Reviews - Cochrane Central Register of Controlled Trials September 2022, Embase 1974 to 2022 November 03, Ovid MEDLINE(R) and Epub Ahead of Print, In-Process, In-Data-Review & Other Non-Indexed Citations, Daily and Versions 1946 to November 03, 2022
Search Strategy:

#	Searches	Results
1	exp Natural Language Processing/	14464
2	exp Machine Learning/	395353
3	exp neural networks, computer/	130114
4	(BERT or "Bidirectional Encoder Representations from Transformers" or "Bio-BERT" or BioWordVec or "Clinical concept extraction" or "convolutional neural network*" or "coreference resolution" or "co-reference resolution" or "Deep Learning" or "extraction framework*" or "Gated Recurrent Unit*" or GRU or "Information extraction" or "Language Model" or "Long Short-Term Memory" or LSTM or "Machine Learning" or "medical language processing" or "Named entity extraction" or "Named entity recognition" or "Natural Language Processing" or "Neural Network*" or NLP or "Recurrent Neural Network*" or "Relation extraction" or RNN or "support vector machine*" or SVM or "Symptom extraction" or "text analysis" or "Text mining" or tree or "Word embeddings").ti,ab,kf.	660768
5	1 or 2 or 3 or 4	862073
6	exp Thyroid Diseases/	424037
7	exp Thyroid Gland/	111946
8	("basedow disease" or "basedows disease" or "Euthyroid Sick Syndrome*" or Goiter or "Graves Disease" or "Graves Ophthalmopathy" or "Hashimoto Disease" or hyperthyroid* or Hyperthyroxinemia or hypothyreoidism* or hypothyreosis or hypothyroid* or hypothyrosis or Myxedema or "sipple disease*" or "sipple syndrome*" or Thyroid* or Thyroiditis or Thyrotoxicosis or "tsh deficienc*").ti,ab,kf.	574247
9	6 or 7 or 8	664058
10	5 and 9	3938
11	limit 10 to english language	3824
12	limit 10 to no language specified	2
13	11 or 12	3826
14	limit 13 to yr="2012 -Current"	3255
15	(case adj3 report).mp,pt.	3447380
16	14 not 15	3123
17	review.pt,ti.	6683671
18	16 not 17	2874
19	limit 18 to (conference abstract or editorial or erratum or note or addresses or autobiography or bibliography or biography or blogs or comment or dictionary or	603

	directory or interactive tutorial or interview or lectures or legal cases or legislation or news or newspaper article or overall or patient education handout or periodical index or portraits or published erratum or video-audio media or webcasts) [Limit not valid in CCTR,Embase,Ovid MEDLINE(R),Ovid MEDLINE(R) Daily Update,Ovid MEDLINE(R) PubMed not MEDLINE,Ovid MEDLINE(R) In-Process,Ovid MEDLINE(R) Publisher; records were retained]	
20	18 not 19	2271
21	remove duplicates from 20	1636

Scopus

1	TITLE-ABS-KEY(BERT OR "Bidirectional Encoder Representations from Transformers" OR "Bio-BERT" OR BioWordVec OR "Clinical concept extraction" OR "convolutional neural network*" OR "coreference resolution" OR "co-reference resolution" OR "Deep Learning" OR "extraction framework*" OR "Gated Recurrent Unit*" OR GRU OR "Information extraction" OR "Language Model" OR "Long Short-Term Memory" OR LSTM OR "Machine Learning" OR "medical language processing" OR "Named entity extraction" OR "Named entity recognition" OR "Natural Language Processing" OR "Neural Network*" OR NLP OR "Recurrent Neural Network*" OR "Relation extraction" OR RNN OR "support vector machine*" OR SVM OR "Symptom extraction" OR "text analysis" OR "Text mining" OR tree OR "Word embeddings")
2	TITLE-ABS-KEY("basedow disease" OR "basedows disease" OR "Euthyroid Sick Syndrome*" OR Goiter OR "Graves Disease" OR "Graves Ophthalmopathy" OR "Hashimoto Disease" OR hyperthyroid* OR Hyperthyroxinemia OR hypothyreoidism* OR hypothyreosis OR hypothyroid* OR hypothyrosis OR Myxedema OR "sipple disease*" OR "sipple syndrome*" OR Thyroid* OR Thyroiditis OR Thyrotoxicosis OR "tsh deficienc*")
3	PUBYEAR AFT 2011 AND LANGUAGE(english)
4	1 and 2 and 3
5	TITLE-ABS-KEY(case W/3 report)
6	4 and not 5
7	TITLE(review)
8	6 and not 7
9	DOCTYPE(ab) OR DOCTYPE(ed) OR DOCTYPE(bk) OR DOCTYPE(er) OR DOCTYPE(no) OR DOCTYPE(sh)
10	8 and not 9
11	INDEX(embase) OR INDEX(medline) OR PMID(0* OR 1* OR 2* OR 3* OR 4* OR 5* OR 6* OR 7* OR 8* OR 9*)
12	10 and not 11
